# Tattoo-induced psoriasis

**Published:** 2014

**Authors:** OA Orzan, LG Popa, ES Vexler, I Olaru, VM Voiculescu, RS Bumbăcea

**Affiliations:** * ”Carol Davila” University of Medicine and Pharmacy, Bucharest, Romania; ** Department of Dermatology, ”Elias” Emergency Hospital, Bucharest, Romania

**Keywords:** Koebner phenomenon, isomorphic phenomenon, koebnerization, psoriatic lesions, tattoos

## Abstract

Koebner phenomenon represents the development of several inflammatory skin lesions (psoriasis, lichen planus, vitiligo, etc.) in uninvolved skin following various traumatic insults. The case of a 27-year-old male patient with scalp psoriasis who was referred to our clinic for generalized psoriatic lesions developed two weeks after tattooing his skin at the age of 18 was presented; the case illustrated the possibility of Koebner phenomenon induced by skin tattooing in patients with psoriasis.

## Introduction

Psoriasis is a chronic inflammatory skin disease with an increased epidermal proliferation and it is usually characterized by erythematous, sharp-bordered lesions with silvery scale that can occur in various sites of the body. Although many different factors can trigger the appearance of psoriatic lesions, a certain cause has not yet been established [**1**]. In patients with this condition, de novo psoriatic lesions often occur at the site of cutaneous trauma. The appearance of these isomorphic lesions is known as the Koebner phenomenon and it occurs in about 25% of people with psoriasis after various traumatic injuries, including tattooing [**2**].

## Case report

A 27-year-old patient was referred to our clinic with erythematosus scaly plaques, disseminated on scalp, trunk and upper limbs. These lesions occurred at the age of 18, two weeks after his first tattooing. Four years prior to this event, the patient was diagnosed with scalp psoriasis.

**Fig. 1:**
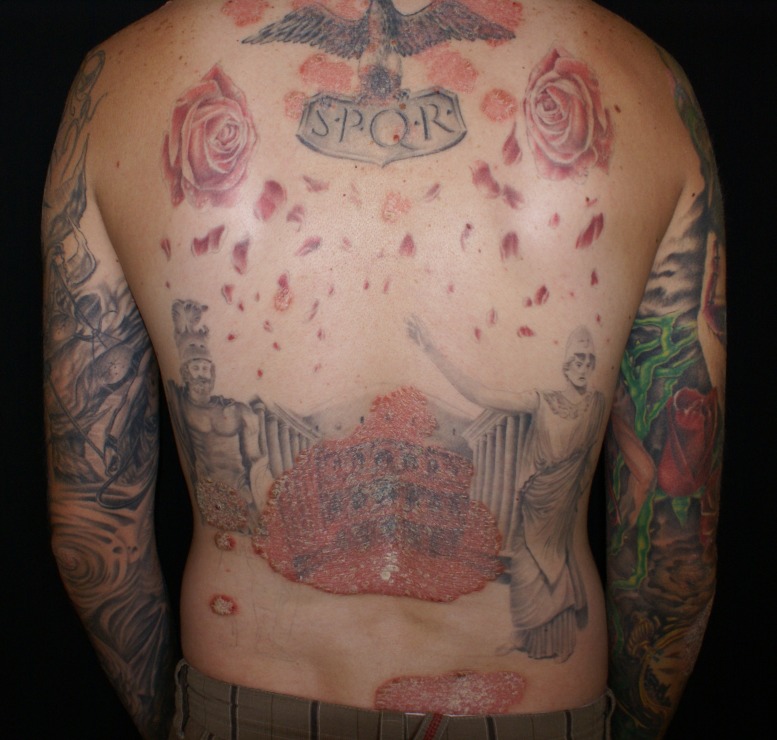
Erythematous plaques covered by silvery white scales on posterior thorax

**Fig. 2:**
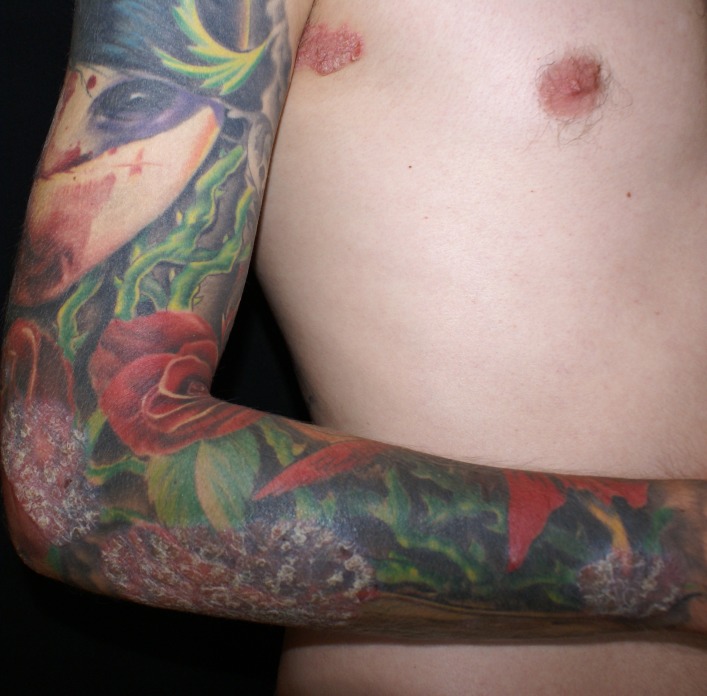
Psoriatic lesions on the tattooed skin

General clinical examination was within the normal range. Local clinical examination revealed multiple well-defined erythematosus plaques, 5 to 25 cm in diameter, covered by thick silvery scales, on the scalp, thorax and upper limbs (**Fig. 1**,**2**). The lesions were located both on normal and on tattooed skin. 

**Fig. 3:**
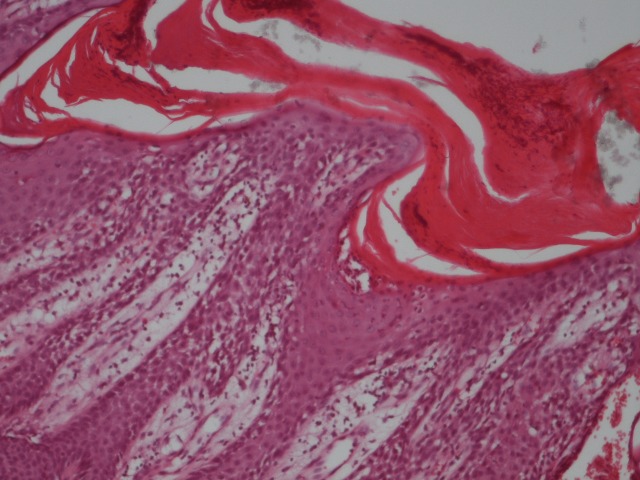
Histopathological exam - Munro's microabscesses and elongated rete ridges

Common laboratory tests were within the normal range. A skin biopsy was taken from a plaque situated on the posterior thorax. The histological examination revealed marked hyperkeratosis, parakeratosis, Munro’s microabscesses, hypo and agranulocytosis, acanthosis and epidermal elongation of the rete ridges (**Fig. 3**).

**Fig. 4:**
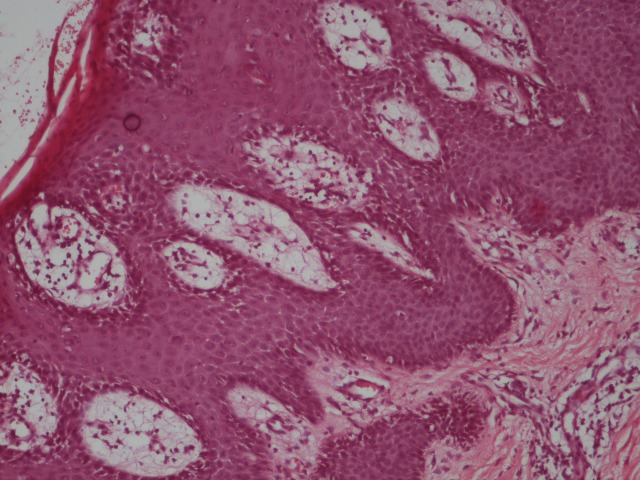
Histopathological exam - Parakeratosis, turgid capillaries and dermal inflammatory infiltrate

A moderate lymphocytic inflammatory infiltrate and frequent dilated capillaries were observed in the papillary dermis (**Fig. 4**). Based on clinical and histological findings the diagnosis of psoriasis vulgaris was made.

Due to the severity of the disease (PASI= 19), local and systemic treatment with Methotrexate (15 kg weekly) was initiated with a favorable evolution. The patient refused phototherapy pretending this could affect the colors of his tattoos.

The patient was followed-up for clinical and biological parameters.

## Discussions

In 1877, the German dermatologist Heinrich Koebner formulated the first definition of Koebner phenomenon by describing the appearance of psoriatic lesions in uninvolved skin of psoriatic patients following local trauma [**3**]. At present, the definition of this phenomenon has been extended, and it also refers to the appearance of lesions along a site of injury even in people without a pre-existing dermatosis [**2**]. Koebner phenomenon, also known as isomorphic response, is seen in several other skin diseases, such as lichen planus and vitiligo. Some authors consider that a variety of other diseases including warts and molluscum contagiosum may koebnerize. However, in these conditions, there is not a “true” koebnerization as the occurrence of new lesions due to the seeding of an infectious organism at the site of trauma. “True” koebnerization refers to those diseases in which the pathogenesis of development of new lesions is identical with that of the main condition (psoriasis, lichen planus, vitiligo) [**4**].

Koebner phenomenon occurs in about 25% of people with psoriasis after various traumatic injuries, especially in the unstable period of the disease [**4**]. The period from the injury to the appearance of new skin lesions is generally between 10 and 20 days, but may range from 3 days to 2 years [**2**]. Various provoking factors have been reported to induce new psoriasis lesions including physical trauma, burns, friction, insect bites, surgical incision, allergic and irritant reactions, radiation exposure, medications and needle acupuncture trauma [**2**,**5**]. Another factor that can induce the isomorphic phenomenon in patients with psoriasis could be the trauma produced by tattooing the skin. Despite the wide popularity of the tattoos, there are few reports in literature of tattoo-induced psoriasis [**6**-**8**]. The relation between the tattoo “trauma” and psoriasis is still controversial with some authors considering that the occurrence of new lesions after this kind of trauma could be just the normal course of the disease. 

In our case, the patient was diagnosed with scalp psoriasis several years prior to this event and he developed generalized lesions of psoriasis 2 weeks after tattooing. The tattoo procedure could have been the provocative factor for psoriasis. There are the 2 weeks onset interval in favor of this scenario and the localization of the psoriatic lesions on the tattooed skin. During the process of tattooing, the dye penetrates the papillary and upper part of the reticular dermis near blood and lymphatic vessels. It is difficult to determine whether the provocative factor for the Koebner phenomenon was the repeated needle trauma as described in acupuncture-induced psoriasis [**5**] or the tattoo ink immune response.

Even though the pathophysiology of the Koebner phenomenon has not been entirely elucidated, it was demonstrated that nerve growth factor (NGF) influences the key pathological events in psoriasis and Koebner phenomenon: keratinocyte proliferation, angiogenesis and T-cell activation [**9**]. In a developing a psoriatic lesion, the up-regulation of NGF together with keratinocyte proliferation are early events and precede epidermotropism of T lymphocytes; keratinocytes in patients with psoriasis are primed to produce elevated levels of NGF [**9**].

## Conclusion

In conclusion, Koebner phenomenon, first described in psoriatic patients, may occur in several other skin diseases and may be induced by various types of triggers, tattooing the skin being one of them [**9**]. Although the isomorphic phenomenon is a rather common one (it occurs in 25% of the patients with psoriasis), the etiology and specific mechanism underlying it have not been completely elucidated [**2**].

With the increasing popularity of tattoos, further research into the immunologic interface of psoriatic precipitators is needed so that the at-risk patients may be aware on their vulnerability to complications secondary to this procedure. Meanwhile, people with psoriasis must weigh the potential of inducing new lesions with the desire to express themselves through body art. 
